# High Hardness, Excellent Hydrophobicity, and Favorable Corrosion Resistance of Plasma-Sprayed FeCrMoSi Amorphous Coatings on 304 Stainless Steel

**DOI:** 10.3390/molecules28186718

**Published:** 2023-09-20

**Authors:** Jiacheng Zhong, Beirui Hou, Wenmin Zhang, Shitao Zhang, Yuantao Zhao, Chunwang Zhao, Wenge Li

**Affiliations:** 1 School of Materials Science and Hydrogen Energy, Foshan University, Foshan 528000, China; zjc160403201@163.com (J.Z.); hbr9396@163.com (B.H.); 15924846452@163.com (W.Z.); 2 Merchant Marine College, Shanghai Maritime University, Shanghai 201306, China; zstcameron@sina.com (S.Z.); zhaoyt@shmtu.edu.cn (Y.Z.); 3 Guangdong Key Laboratory for Hydrogen Energy Technologies, Foshan 528000, China

**Keywords:** FeCrMoSi amorphous coating, corrosion resistance, hardness, hydrophobicity, bonding strength

## Abstract

The FeCrMoSi amorphous coatings were fabricated on the surface of a 304 stainless steel (SS) base material using atmospheric plasma spraying. A comprehensive investigation was carried out to evaluate the structure, morphology, adhesion to base material, hardness, hydrophobicity, interfacial contact resistance, and corrosion resistance of the coatings. The results show a remarkable hardness of 1180.1 HV, a strong bond strength of up to 64.3 N/mm^2^, and excellent hydrophobicity with a water contact angle reaching 141.2°. Additionally, in an acidic environment with fluoride ions (0.5 M H_2_SO_4_ + 2 ppm HF, 80 °C), the FeCrMoSi amorphous coating demonstrated superior corrosion resistance compared with 304 SS while maintaining similar electroconductibility. Detailed analysis of the structural characteristics and corrosion resistance of FeCrMoSi amorphous coatings provided valuable insights into their mechanics. These promising results signify a bright future for FeCrMoSi amorphous coatings in various industrial sectors, including transportation, petroleum, and electric power industries.

## 1. Introduction

304 Stainless Steel (SS) is widely used in the manufacture of equipment and machine parts that require superior all-round performance [1,2]. One of its most notable properties is excellent corrosion resistance. This is due to the high Cr content, which forms a stable Cr oxide layer on the surface of the 304 SS [3,4]. This not only provides effective protection against water, oxygen, and many chemicals but also makes it the material of choice in wet, acidic, alkaline, and corrosive environments [5]. It also has appropriate strength and temperature resistance. This allows it to be used in a wide range of applications including construction, manufacturing, chemical, and high temperature applications [6]. Despite its superior corrosion resistance and durability, the Cr oxide layer of 304 SS may not be sufficient to provide adequate protection in some extreme corrosive environments [7,8]. Therefore, it is necessary to apply anticorrosion coatings to the surface of 304 SS to increase its corrosion resistance [9]. Corrosion-resistant coatings play a crucial role in safeguarding base materials from corrosion or retarding the rate of corrosion. For different corrosive environments, a wide array of such coatings have been developed. For example, Meghwal et al. found that AlCoCrFeNi high-entropy alloy coating exhibited better corrosion resistance than that of 316 L SS in seawater [10]. Similarly, Duran et al. discovered that poly(N-vinyl carbazole) films can provide anodic protection and reduce the corrosion rate of 304 SS by six times in sulfuric acid solution [11]. Additionally, Sun et al. deposited Cr–Al coating on 304 SS and found it to have good corrosion resistance under static lead–bismuth eutectic [12].

Amorphous alloy coatings are highly popular and cost-effective choices for corrosion resistance coatings. They can be easily prepared by thermal spraying techniques. In this process, raw material powder is heated to a molten or semi-molten state, then accelerated using a high-speed flame to coat the surface of the base material. In most cases, thermal spray coatings demonstrate remarkable protection to the base material by providing favorable resistance to corrosion, wear, and oxidation. Currently, thermal spraying technologies include flame spraying [13], arc spraying [14], plasma spraying [15,16], supersonic flame spraying [17], and explosive spraying [18]. Among these technologies, plasma spraying, which has been widely applied to protect metal base materials in industrial production by improving their corrosion resistance, wear resistance, and microhardness, is frequently used [19]. For example, Zhao et al. used atmospheric plasma spraying (APS) to deposit a Mo-based amorphous nanocrystalline coating on the surface of ordinary carbon steel base material. They found that the hardness and the abrasiveness of the carbon steel with the coating were significantly increased compared with the base material [20]. The fabrication of amorphous coatings by APS cannot only achieve large area construction, simple operation, and low cost but also does not lose the intrinsic excellent properties of amorphous raw materials. Therefore, APS is a simple and efficient way to fabricate amorphous coatings.

Fe-based amorphous coatings have also emerged as a cost-effective solution, offering high hardness and excellent wear resistance [21,22]. For instance, Hamid et al. deposited Fe-based amorphous coatings using high-velocity oxygen fuel (HVOF) spraying, showcasing remarkable ability to resist friction and wear performance [23]. Meanwhile, Cao et al. fabricated a crack-free Fe-based amorphous coating on a 304 SS base material using laser processing technology. Their findings suggested that the amorphous phase of the coating and its optimized microstructure significantly increased the hardness with a favorable effect on the coefficient of friction and wear rate [24]. Qiao et al. fabricated a superhydrophobic Fe-based amorphous coating using APS on a Q235 steel base material and demonstrated that the coating had an ultra-high microhardness and good antiwear properties [25]. These previous studies confirmed that Fe-based amorphous coating could be simply fabricated to provide good protection to the base material. However, the corrosion resistance and corresponding mechanism of Fe-based amorphous coatings to a harsh acidic environment remain unclear to date.

This study focuses on the fabrication of FeCrMoSi amorphous coatings through APS on a 304 SS base material. A comprehensive investigation was conducted, covering the structure, morphology, hydrophobicity, bonding strength to the base material, and corrosion resistance in a mixed sulfuric acid and hydrofluoric solution at 80 °C, as well as the interface contact resistance of the coatings.

## 2. Results and Discussion

### 2.1. Structure and Morphology

**Figure 1:**
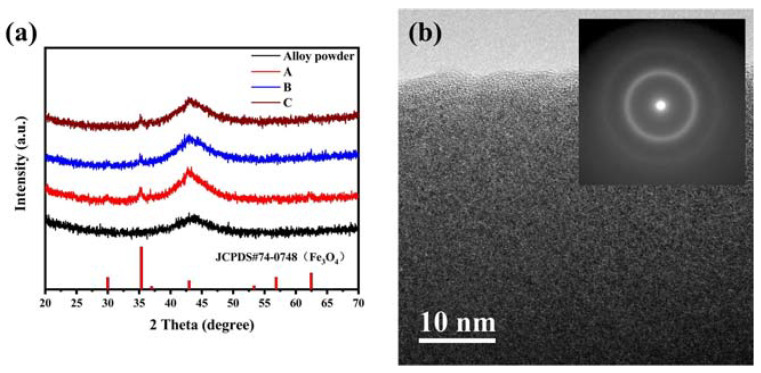
(**a**) XRD patterns of FeCrMoSi alloy powder raw materials and as-fabricated FeCrMoSi amorphous coatings; (**b**) TEM image and the corresponding SAED pattern (in set).

The phase structure of FeCrMoSi alloy raw materials powder and as-fabricated FeCrMoSi amorphous coatings were examined using X-ray diffraction (XRD); the XRD patterns are presented in Figure 1a. The XRD pattern of the raw materials exhibited a single broadened and diffused diffraction peak at approximately 43°. This indicates that the FeCrMoSi alloy raw materials are in a completely amorphous state. Similarly, each XRD pattern of the coatings also exhibited a broadened and diffused diffraction peak around 43°, consistent with that of the raw materials, further confirming that the coatings are predominantly in an amorphous state. However, in each XRD pattern of the FeCrMoSi amorphous coatings, a small sharp peak at about 35°, which could be attributed to Fe_3_O_4_, was observed [25,26]. This finding suggests the occurrence of a certain oxidation reaction during the fabrication of the coatings by APS. In addition, the bright field transmission electron microscopy (TEM) images of FeCrMoSi amorphous coatings show a typical amorphous morphology (Figure 1b) and the corresponding selected area electron diffraction (SAED) pattern only shows a typical diffuse ring. Consequently, both XRD patterns and TEM observation demonstrated that FeCrMoSi amorphous coatings with high amorphous phase content may contain a very small amount of crystalline structure.

**Figure 2:**
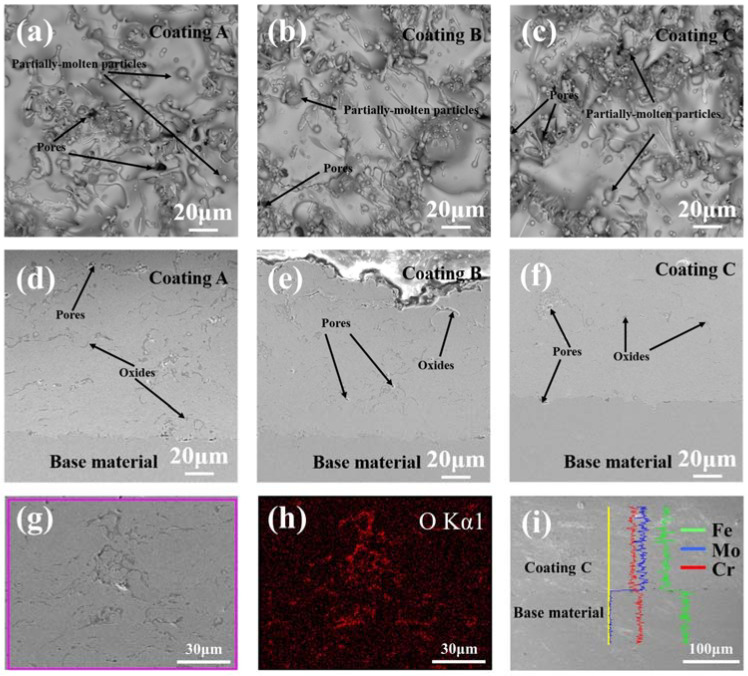
SEM images of FeCrMoSi amorphous coatings: surface morphologies of coatings (**a**) A, (**b**) B, and (**c**) C; cross-section morphologies of coatings (**d**) A, (**e**) B, and (**f**) C; (**g**) surface area of coating C used for EDS mapping and (**h**) corresponding EDS mapping of O element; (**i**) EDS line scanning result from coating to base material.

The surface morphology of the coatings and the interface between the coatings and the base material were observed using scanning electron microscopy (SEM); the images are depicted in Figure 2. Each coating (Figure 2a–c) exhibited partially molten particles and micropores. The number of partially molten particles on the surface gradually decreased with the increase in applied current during APS. Among the coatings, coating C displayed the highest degree of melting under a current of 600 A. The high-temperature molten FeCrMoSi alloy particles experienced rapid impact on the surface of the 304 SS base material during APS. Some particles were fully deformed, forming a typical layered structure and resulting in a certain smooth surface, meanwhile others remained spherical after APS, leading to incomplete packing between molten flat particles and the appearance of a small number of pores. Additionally, the cold shrinkage of molten flat particles during solidification may have contributed to the formation of some pores [24]. Figure 2d–f show the cross-sectional morphologies of the coatings. With increasing current, the presence of pores in the coatings reduced. However, even at the maximum current, some pores persisted between the coatings and the 304 SS base material. These pores can be found to be oxygen-rich by X-ray energy dispersive spectroscope (EDS) mapping (Figure 2g,h). These phenomena may be attributed to slight oxidation in air [27], considering that the raw materials needed to be heated to their melting temperature during APS. Subsequently, the chemical composition of the FeCrMoSi amorphous coatings was examined using EDS and the results are summarized in Table 1. The chemical composition of the as-fabricated coatings generally reflects that of the FeCrMoSi alloy powder raw materials, with slight oxidation observed. The elemental distribution of coating C and the base material was measured by EDS line scanning and the results are shown in Figure 2i. It is seen that the coating mainly contains the elements Fe, Cr, and Mo and the distribution of these three elements is uniform. Comparing to 304 SS base materials, the Cr content in the coating is slightly lower than that of 304 SS. The Fe content in the coating is largely lower than that of 304 SS. Meanwhile, 304 SS base material does not contain Mo element. Previous studies have shown that adding Mo to an alloy can form a protective film on the surface, which can effectively prevent further corrosion inside the alloy [28]. Furthermore, the migration or diffusion of the alloying elements from the base material to the coating and vice versa may occur during the coating process. However, the current EDS line scanning does not detect this phenomenon, which can be further studied in the future.

### 2.2. Bonding Strength

**Figure 3:**
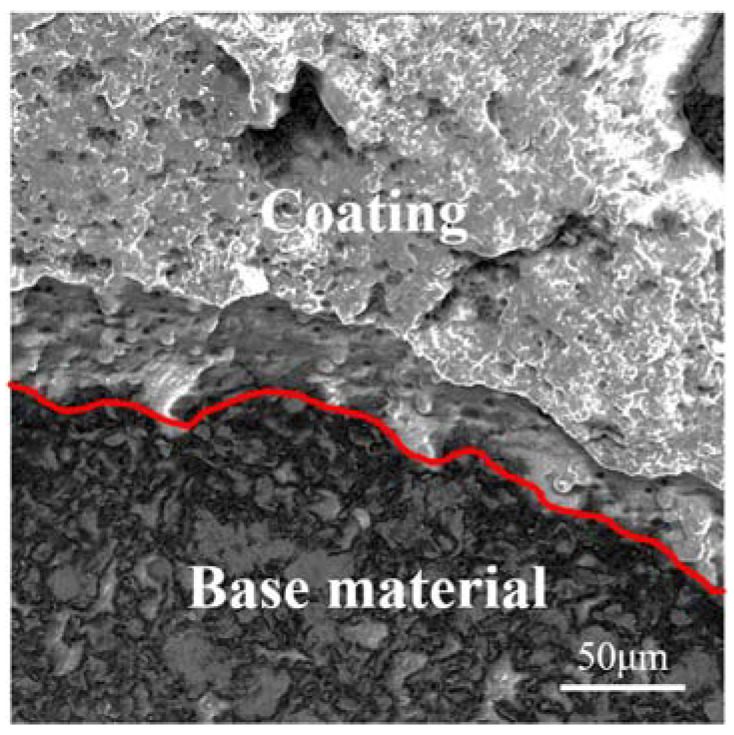
SEM image of the fracture surface of the coating and base material after bonding strength test.

The bonding strengths between the coatings and the 304 SS base material were measured using the tensile test and the results are summarized in Table 2. Coatings A, B, and C exhibited bonding strengths of 64.3, 61.6, and 53.1 N/mm^2^, respectively. For comparison, Zhang et al. prepared Fe-based amorphous coatings on WE43 magnesium alloy using HVOF spraying, with bonding strength reaching 56 MPa [29]. In another study, Zhang et al. prepared laminar coatings comprising multi-amorphous Fe48Mo14C15Y2C15B6 layers and a crystalline NiCrAl layer using the HVOF technique and the bonding strength of those coatings was only 40 MPa [30]. In our work, the bonding strength of the coatings reached up to 64.3 N/mm^2^, indicating a strong bonding state between the coating and the base material. SEM images of the fracture surface of the coating and the base material after the bonding strength test revealed some flat FeCrMoSi alloy particles still attached to the base material, demonstrating a robust binding force between them (Figure 3). Prior to spraying, the base materials were sandblasted to increase the surface roughness, thus enhancing the bonding strength between the coatings and the base material. Notably, FeCrMoSi amorphous alloy particles experienced rapid heating in atmospheric plasma, resulting in inevitable oxidation [31]. Subsequently, these molten particles were sequentially sprayed onto the base material’s surface, forming laminar patches with oxygenated interfaces on the 304 SS base material (Figure 2). Based on the above observations and tests, the bonding and failure mechanism of the as-prepared FeCrMoSi amorphous coatings can be analyzed as follows: During the initial APS, the sprayed FeCrMoSi alloy particles combine with the base material and leave cavities between the particles, resulting in a porous structure on the coating surfaces. As atomic diffusion is sufficiently active, the particles undergo plastic deformation, leading to an improved engagement between the coatings and the base material. When the coating is subjected to tensile force, cracks continue to extend along the defective areas due to the presence of some oxides and pores. Consequently, when the extended cracks contact each other, the fracture occurs in the weakest region inside the coating.

### 2.3. Hardness

The Vickers hardness values of the coatings and the 304 SS base material are summarized in Table 3. Evidently, each FeCrMoSi amorphous coating exhibits a hardness approximately five times greater than that of the 304 SS base material. This significant increase in hardness could be attributed to the presence of Mo element in the coatings, which has been known to substantially enhance material hardness [32]. The remarkable hardness of the coatings bodes well for their wear resistance, offering effective protection to the base material [22,23,24].

### 2.4. Hydrophobicity

**Figure 4:**
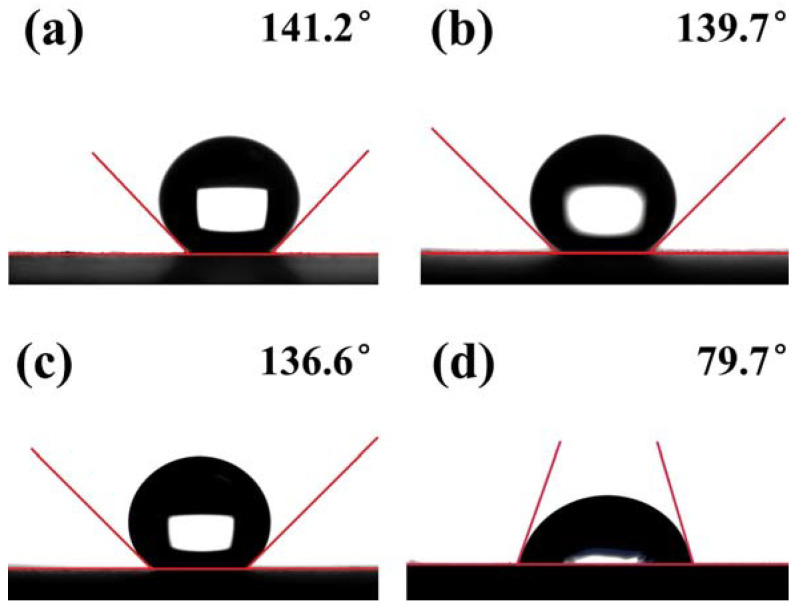
WCA of (**a**–**c**) FeCrMoSi amorphous coatings and (**d**) 304 SS.

Hydrophobicity was characterized by measuring the water contact angle (WCA) of the FeCrMoSi amorphous coatings and the 304 SS base material, as depicted in Figure 4. Remarkably, the FeCrMoSi amorphous coatings exhibited excellent hydrophobicity, with WCAs ranging from 136.6° to 141.2°. In contrast, the WCA of the 304 SS was only 77.5°. The hydrophobicity of the FeCrMoSi amorphous coatings surpassed that of the NiCrBSi coating, which recorded a WCA of 128° [33], and even exceeded the chromium-doped titanium nitride film, with a WCA of only 115.86° [34].

**Figure 5:**
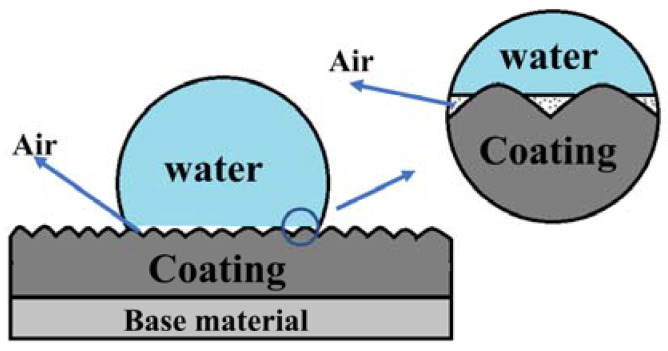
Schematic illustration for the hydrophobic mechanism of FeCrMoSi amorphous coatings.

The hydrophobicity of a coating is closely related to its surface morphology [35] and the direct attraction of water droplets to the surface materials, which is minimized due to the lower surface energy. Generally, greater surface roughness results in improved hydrophobicity [36]. Models used to describe hydrophobic states can be divided into two types. The first is called the Wenzel state, wherein water droplets completely penetrate the roughness [37]. The second is the Cassie–Baxter state, which assumes that only the upper region of the rough surface is in contact with water and an air pocket exists between them [38]. For the FeCrMoSi amorphous coatings in this study, the hydrophobicity of the FeCrMoSi amorphous coatings suggests that the water droplets cannot fully penetrate any part of the coating surface and can only form an “air wall” at the groove, which traps the air in the groove, creating a slight “air cushion” effect [39]. This effect supports the water droplets, thereby increasing the hydrophobicity of the coating (Figure 5). According to SEM observations, numerous small humps are present on the surface of the coatings (Figure 3), which likely result from the partial melting and simultaneous splashing of the molten or semi-molten alloy particles during APS [40].

### 2.5. Corrosion Resistance

**Figure 6:**
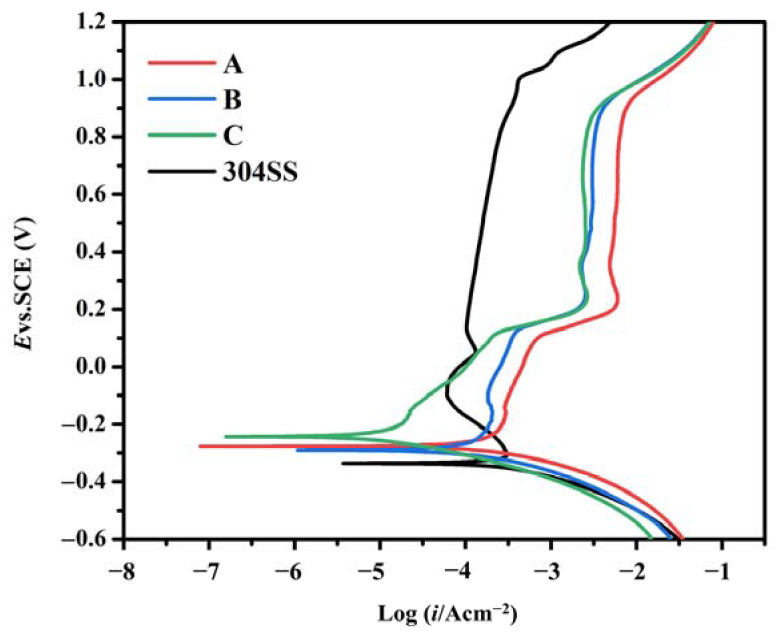
Potentiodynamic polarization curves of FeCrMoSi amorphous coatings and 304 SS.

The potentiodynamic polarization curves of the FeCrMoSi amorphous coatings and the 304 SS base material in a hydrogen-passing sulfuric acid and hydrofluoric mixed solution (0.5 M H_2_SO_4_ + 2 ppm HF, 80 °C) are presented in Figure 6, with the corresponding electrochemical parameters summarized in Table 4. The results demonstrate an active–passive transition for the 304 SS base material. As the potential increases, the anodic polarization curve of 304 SS forms a stable passivation region at 0.09–1 V. However, as the anode polarization potential further increases, the anode current density rises rapidly, indicating that the passivation film begins to break down, losing its passivation ability in the 1–1.2 V range. In contrast, the FeCrMoSi amorphous coatings exhibit superior corrosion resistance compared to 304 SS, evidenced by their lower corrosion current density (I_corr_) and higher corrosion potential value (E_corr_). The E_corr_ of the FeCrMoSi amorphous coatings is approximately 90 mV greater than that of the 304 SS. Particularly noteworthy is the fact that the I_corr_ of the FeCrMoSi amorphous coatings is lower than that of the 304 SS base material by one to two orders of magnitude. According to the potentiodynamic polarization curves (Figure 6), the FeCrMoSi amorphous coatings form a passivation film at lower current densities, remaining stable at higher potentials. Coatings A and B exhibit a slow activation process before passivation. Once the E_corr_ reaches approximately 0.15 V, the I_corr_ increases rapidly until reaching the equilibrium state of active dissolution and passivation. However, the I_corr_ of coating C increases rapidly before passivation, and passivation begins near 0.25 V. This active reaction is primarily attributed to the dispersed distribution of particles, oxides, and pores in the coating. Such structural defects often become sites of initial corrosion, leading to a swift increase in the early I_corr_ of anodic polarization until the appearance of a passivation state.

**Figure 7:**
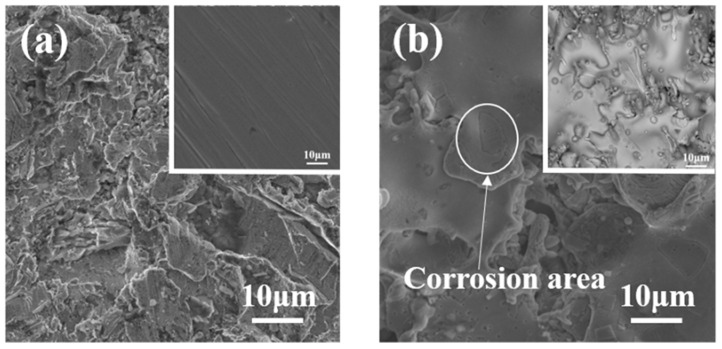
SEM images of (**a**) 304 SS and (**b**) coating C after corrosion resistance test. Insets are corresponding SEM images before corrosion resistance test.

The surface morphologies of a typical FeCrMoSi amorphous coating C and the 304 SS base material before and after the corrosion resistance test were observed using SEM, as depicted in Figure 7. Clearly, the 304 SS suffered severe corrosion damage, with the surface covered in corrosion products and corrosion pits (Figure 7a). In contrast, the overall morphology of the FeCrMoSi amorphous coating did not change significantly, with only a small number of corrosion pits observed on the surface (Figure 7b). These SEM observations further confirm that the FeCrMoSi amorphous coating exhibits superior corrosion resistance compared with 304 SS.

The chemical state and composition of the corrosion products of the typical FeCrMoSi amorphous coating C before and after the corrosion resistance test were investigated using X-ray photoelectron spectroscopy (XPS). The original XPS data were initially calibrated with carbon 1 s (284.6 eV). Subsequently, XPS data analysis was carried out using the XPS Peak 4.1 program and a Shirley function was employed to subtract the background. The Fe 2p, Cr 2p, and Mo 3d core-level spectra were fitted with Lorentzian–Gaussian curves [41]. For the Fe 2p_1/2_, Fe 2p_3/2_, Cr 2p_1/2_, and Cr 2p_3/2_ core-level spectra, a 1:2 ratio was applied in the curve fitting of all the Fe 2p and Cr 2p XPS spectra. Similarly, the Mo 3d_3/2_ and Mo 3d_5/2_ signal areas had a 2:3 ratio which was applied in the curve fitting of all the Mo 3d XPS spectra.

**Figure 8:**
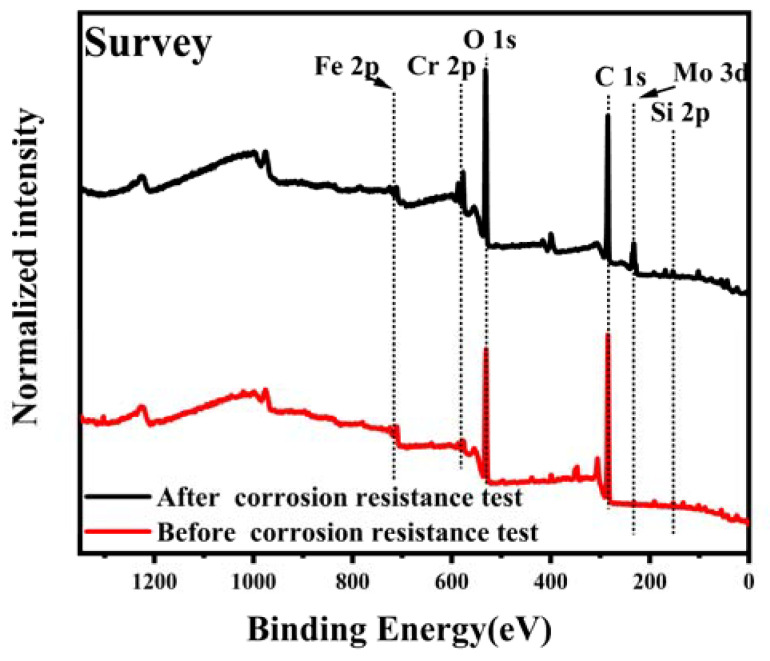
XPS survey spectrum of coating C before and after corrosion resistance test.

**Figure 9:**
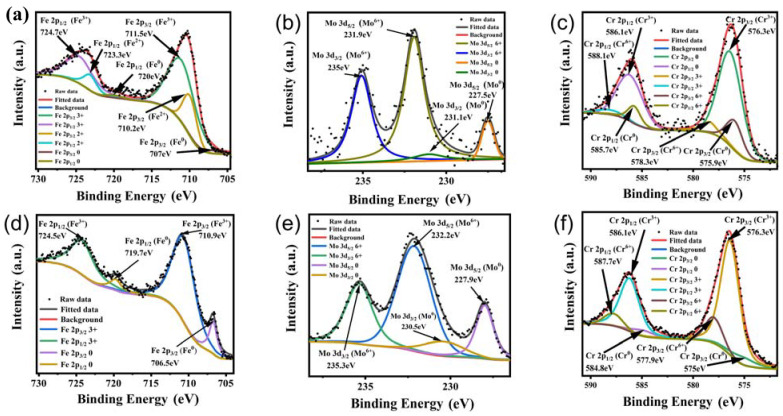
XPS core-level spectra of (**a**) Fe 2p, (**b**) Mo 3d, and (**c**) Cr 2p before and (**d**) Fe 2p, (**e**) Mo 3d, and (**f**) Cr 2p after corrosion resistance test of coating C.

The XPS survey spectrum (Figure 8) clearly indicates that in addition to adventitious carbon, some main elements include Fe, Mo, Cr, Si, and O. The XPS core-level spectra of Fe before the corrosion resistance test can be identified to be composed of Fe 2p_1/2_ and Fe 2p_3/2_ peaks of Fe^0^, Fe^2+^, and Fe^3+^ states [42], as depicted in Figure 9a. The presence of Fe^2+^ and Fe^3+^ indicates that a small amount of Fe has been oxidized during APS. This reaction corresponds to the appearance of Fe_3_O_4_ in the XRD patterns (Figure 1), which is composed of FeO and Fe_2_O_3_ [43]. The XPS core-level spectra of Mo before the corrosion resistance test can be identified to be composed of Mo 3d_3/2_ and Mo 3d_5/2_ peaks of Mo^0^ and Mo^6+^ states [44,45], as displayed in Figure 9b. The presence of Mo6+ indicates that a small amount of Mo has been oxidized during APS. The XPS core-level spectra of Cr before the corrosion resistance test can be identified to be composed of Cr 2p_1/2_ and Cr 2p_3/2_ peaks of Cr^0^, Cr^3+^, and Cr^6+^ states [46], as presented in Figure 9c. The presence of Cr^3+^ indicates that a small amount of Cr has been oxidized during APS.

The XPS core-level spectra of Fe after the corrosion resistance test reveal the presence of Fe 2p_1/2_ and Fe 2p_3/2_ peaks corresponding to Fe^0^ and Fe^3+^ states, as marked in Figure 9d. In comparison to the XPS core-level spectra of Fe before the corrosion resistance test (Figure 9a), Fe^2+^ ions were not found, indicating that Fe^2+^ is more soluble in the acidic solution (0.5 M H_2_SO_4_ + 2 ppm HF, 80 °C). Similarly, the XPS core-level spectra of Mo after the corrosion resistance test exhibit Mo 3d_3/2_ and Mo 3d_5/2_ peaks corresponding to Mo^0^ and Mo^6+^ states, as marked in Figure 9e. Likewise, the XPS core-level spectra of Cr after the corrosion resistance test show Cr 2p_1/2_ and Cr 2p_3/2_ peaks corresponding to Cr^3+^ states only, as marked in Figure 9f. These spectra are consistent with those before the corrosion resistance test, indicating that the Mo and Cr elements can withstand the corrosion of this specific acidic solution (0.5 M H_2_SO_4_ + 2 ppm HF, 80 °C).

**Figure 10:**
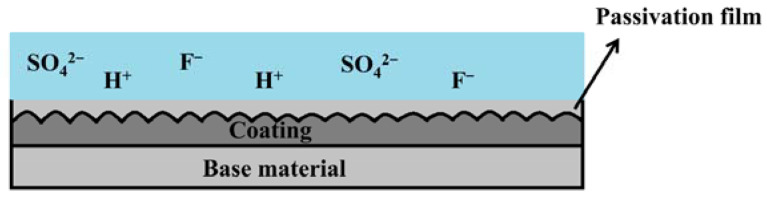
Schematic illustration for the corrosive ions and the passivation film.

Based on the above analysis, the passivation film on the surface of the coating is believed to be composed of oxides of Cr, Fe, and Mo, with the main compounds being Fe_3_O_4_, Cr_2_O_3_, and MoO_3_. These oxides significantly improve the stability of the passivation film and enhance the protective effect of the chromium oxide film [47]. Xia et al. have proposed that hexavalent chromium dissolves in water and increases the corrosion current density, but it possesses a self-healing ability and can promote the formation of dense chromium oxide [48]. The presence of Cr ions is crucial for the corrosion resistance of FeCrMoSi amorphous alloy coatings as they can form chromium hydroxide passivation films. Additionally, Cr reacts with H_2_O to form Cr_2_O_3_, which further enhances its corrosion resistance [49]. This also explains the significant reduction in the intensity of Cr^0^ after corrosion. Moreover, Tian et al. have suggested that MoO_3_ can accumulate on the surface and hinder the diffusion of corrosion ions into the interior (Figure 10), effectively preventing pitting corrosion [43]. Thus, FeCrMoSi amorphous coatings demonstrate better corrosion resistance than 304 SS due to their higher Cr and Mo contents. Furthermore, the chemical composition of the corroded surface of coating C (Table 5) shows that the O content has doubled compared to that of the as-fabricated coating, indicating a substantial production of oxides on the surface of the coating during the corrosion resistance test.

### 2.6. Interfacial Contact Resistance (ICR)

**Figure 11:**
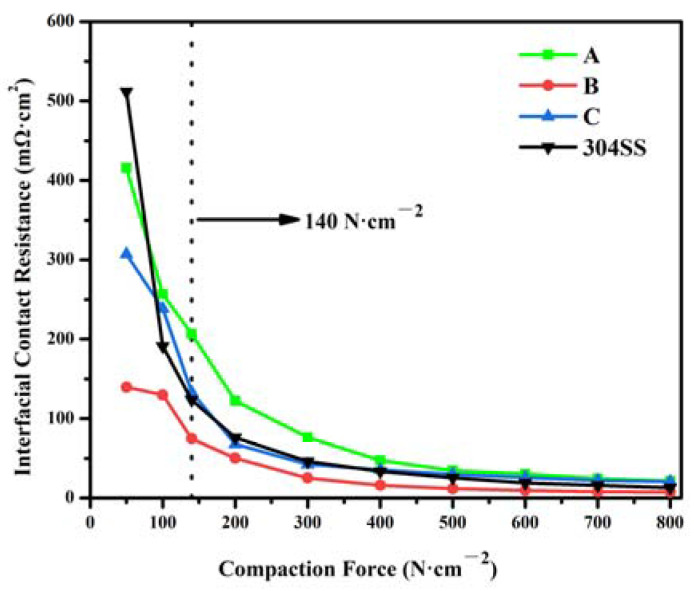
ICR of FeCrMoSi amorphous coatings and 304 SS under 50–800 N/cm^2^ compaction forces.

The electrical conductivity of FeCrMoSi amorphous coatings and 304 SS under different compaction forces was measured using ICR and the results are presented in Figure 11. The ICR decreases with increasing compaction forces, attributed to the increase in effective contact area [45]. Specifically, the ICR values at 140 N/cm^2^ are summarized in Table 6. Coating A exhibits a high ICR with increasing compressive force, indicating low electrical conductivity of the coating. The ICR of coating C is lower than that of coating A. However, the ICR of coating B is significantly lower than that of the 304 SS (123.5 mΩ·cm^2^) [44].

Although the electrical conductivity of FeCrMoSi amorphous coatings is much better than that of 304 SS, the ICR still failed to meet the 2025 U.S. Department of Energy (DOE) target (<10 mΩ·cm^2^) for bipolar plate of hydrogen fuel cell [50]. Further improvement for decreasing the ICR of FeCrMoSi amorphous coatings may be conducted in the future.

## 3. Materials and Methods

**Figure 12:**
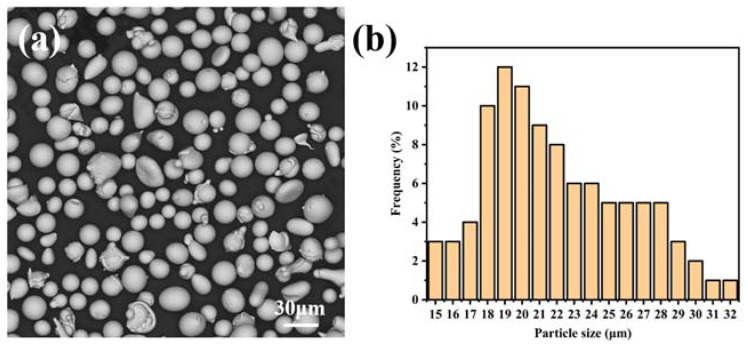
Morphology of FeCrMoSi amorphous alloy powder: (**a**) SEM image; (**b**) particle size.

The feedstocks of FeCrMoSi alloy powder raw materials (Figure 12a) were procured from Sunny Metal Technology Company, Republic of Korea. The majority of the powders are spherical, with diameters ranging from 15–32 μm (Figure 12b). An EDS was employed to analyze the chemical composition of the FeCrMoSi amorphous alloy powders, revealing Fe, Mo, Cr, and Si as the main elements, as shown in Table 7. The base material utilized for this study was 304 SS, with dimensions of 40 × 40 × 2 mm^3^. Prior to APS, the 304 SS base material underwent sandblasting to remove the oxide layer and achieve a rough surface. The FeCrMoSi amorphous coatings were fabricated using a UniCoatPro APS system (Oerlikon, Pfäffikon, Switzerland) with a F4MB gun and the spraying parameters are detailed in Table 8. The coatings produced using low, intermediate, and high spraying currents were labeled as coatings A, B, and C, respectively.

**Figure 13:**
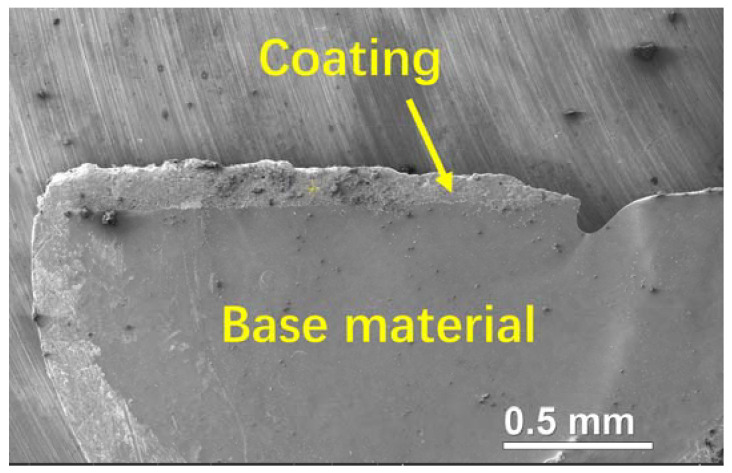
SEM image of the cross-sectional TEM sample.

The surface morphology and chemical composition of the coatings, along with the interface morphology between the coatings and the base materials, were observed and measured using a TM3030 SEM (Hitachi, Tokyo, Japan) equipped with an Swift 3000 EDS (Oxford Instruments, Oxford, UK). The phases of the coatings were examined using a Ultima IV XRD (Rigaku, Tokyo, Japan) at 40 kV and 30 mA, with a scanning speed of 5°/min. Meanwhile, a cross-sectional TEM sample was prepared using a twin-jet electropolisher, as shown in Figure 13. The coating on the surface of the 304 SS base material can be clearly identified, as marked with the arrow. The coating thickness was measured to be about 136 μm. Bright field TEM and selected area electron diffraction images were collected on a Talos F200X S/TEM (Thermo Scientific, Waltham, MA, USA) at an accelerating voltage of 200 kV. WCA was measured using a JC2000D1B video-based optical instrument (Zhongchen, Shanghai, China) with 5 μL deionized water droplets. Each sample was measured 10 times and the average value was recorded. The bonding strength between the coatings and the base material was determined using a Roell Z250 electronic universal testing machine (Zwick, Ulm, Germany) through a pairwise tensile method. The specimens were cylindrical with a diameter of 25.4 mm, tested following ASTM C633-01 Standard Test Method for Adhesion or Cohesion Strength of Thermal Spray Coatings. The Vickers hardness of the coatings and the base material was measured using an HXS-1000 microhardness tester (Shangguang, Shanghai, China) with a load of 100 gf and a holding time of 15 s, following ASTM E384-22 Standard Test Method for Microindentation Hardness of Materials. To minimize measuring errors, 10 indentations were conducted for each sample.

The ICR test [51] entailed positioning carbonized paper and copper plates on both sides of the sample, subjecting it to varying compressive forces via a M350-CT (Testometric, Rochdale, UK) universal testing machine, while recording the resistance with a ZY9858 digital micro-ohmmeter (Zhengyang, Shanghai, China). Each sample underwent three measurements and the average value was recorded.

The corrosion resistance testing was carried out using a three-electrode system comprising a platinum sheet as the counter electrode, a saturated calomel electrode (SCE) as the reference electrode, and the specimen as the working electrode. A CS150M electrochemical workstation (CorrTest, Wuhan, China) was employed with 1 cm^2^ of the sample surface area exposed to the electrolyte. The electrolyte consisted of 0.5 M H_2_SO_4_ + 2 ppm HF. Initially, the samples were immersed in the electrolyte for 1 h while open circuit potential tests were performed. Subsequently, potentiodynamic polarization tests were conducted in the range of −0.6 V to +1.2 V at a scanning speed of 0.2 mV/s. Corrosion potentials and corrosion current densities were obtained through Tafel extrapolation, implemented in the electrochemical analysis software, with Tafel fitting performed in the potential range of ±200 mV around the open circuit potential. The corrosion resistance tests were conducted at 80 °C with H2 bubbles at a gas flow rate of 20 mL/min. After the corrosion resistance test, the surface morphology of each sample was observed again using SEM and the chemical composition of the coatings was re-examined using EDS.

X-ray photoelectron spectroscopy (XPS) was utilized to analyze the chemical composition and chemical state of FeCrMoSi amorphous coatings both before and after the corrosion resistance test. The ESCALAB 250Xi spectrometer (Thermo Scientific, Waltham, MA, USA) was employed for this purpose, maintaining a base pressure below 1.0 × 10^−7^ Pa through a combination of a mechanical pump and a turbo molecular pump. The excitation source was a nonmonochromatic dual anode X-ray source emitting Al Kα photons with an energy of 1486.6 eV, operating at 14.6 kV. During acquisition, the survey spectrum was scanned with a step of 1 eV, followed by sequential acquisition of the O 1s, Fe 2p, Mo 3d, and Cr 2p core-level spectra with a scan step of 0.05 eV. The analyzed area formed a circle with a diameter of 400 μm. To compensate for the charge up effect, a charge neutralizer was employed.

## 4. Conclusions

This paper presents the fabrication and characterization of FeCrMoSi amorphous coatings using atmospheric plasma spraying technology. The coatings were subjected to XRD, SEM, hydrophobicity test, bonding strength test, hardness test, corrosion resistance test, and electroconductibility test. The results reveal that the chemical composition of the plasma-sprayed coatings undergoes only slight changes with variations in current and hydrogen during the fabrication process. Notably, the FeCrMoSi amorphous coatings demonstrate a higher passivation film breakdown potential (~1 V) and exhibit less corrosion damage, indicating superior corrosion resistance compared to 304 SS. Furthermore, the coatings exhibit an impressive hardness of up to 1180.1 HV, which is five times greater than that of the 304 SS, showcasing excellent wear resistance. Additionally, the coatings display excellent hydrophobicity with a water contact angle reaching 141.2°. These unique properties suggest that FeCrMoSi amorphous coatings have the potential to serve as exceptional protective materials in harsh corrosive environments containing H^+^, F^−^, SO_4_^2−^, and H_2_, particularly in marine, hydraulic, and fuel cell applications.

## Figures and Tables

**Table 1:** Chemical composition of FeCrMoSi amorphous coatings (at. %).

Coatings	Mo	Cr	Si	O	Fe
A	17.17	16.26	0.76	8.12	Bal.
B	15.56	15.35	1.56	7.35	Bal.
C	13.38	14.58	1.21	5.21	Bal.

**Table 2:** Bonding strength between the coatings and 304 SS.

Coatings	A	B	C
N/mm^2^	64.3	61.6	53.1

**Table 3:** Vickers hardness of the coatings and 304 SS.

Coatings	Hardness (HV)
A	1180.1 ± 2.3
B	1063.6 ± 1.2
C	1057.4 ± 2.4
304 SS	209.3 ± 1.3

**Table 4:** Electrochemical parameters of FeCrMoSi amorphous coatings and 304 SS.

Coatings	E_corr/_V	I_corr/_A·cm^−2^
A	−0.28	2.8 × 10^−4^ ± 3.1 × 10^−9^
B	−0.29	3.3 × 10^−4^ ± 1.4 × 10^−8^
C	−0.24	1.5 × 10^−5^ ± 1.7 × 10^−11^
304 SS	−0.34	4.1 × 10^−4^ ± 1.2 × 10^−8^

**Table 5:** Chemical composition of coating C after corrosion resistance test.

Elements	Mo	Cr	Si	O	Fe
at. %	12.25	11.12	1.12	15.51	Bal.

**Table 6:** ICR of FeCrMoSi amorphous coatings and 304 SS at 140 N/cm^2^.

Coatings	ICR (mΩ·cm^2^)
A	206.5 ± 1.2
B	74.7 ± 0.7
C	133.3 ± 1.5
304 SS	123.5 ± 3.5

**Table 7:** Chemical composition of FeCrMoSi alloy powder raw materials.

Elements	Mo	Cr	Si	Fe
at. %	18.05	19.51	3.16	Bal.

**Table 8:** Spraying parameters of FeCrMoSi amorphous coatings.

Coatings	Current (A)	Ar (L/min)	H_2_ (L/min)	Feed Rate (g/min)
A	400	45	13	24
B	520	45	5	24
C	600	45	5	24

## Data Availability

Data is contained within the article.
